# Difference in Sun Exposure Habits Between Individuals with High and Low Risk of Skin Cancer

**DOI:** 10.5826/dpc.1104a90

**Published:** 2021-10-01

**Authors:** Oskar Karlsson, Oskar Hagberg, Kari Nielsen, John Paoli, Åsa Ingvar

**Affiliations:** 1 Department of Dermatology, Skåne University Hospital, Lund, Sweden; 2 Institution of Translational Medicine, Lund University, Malmö, Sweden; 3 Department of Clinical Sciences, Lund, Division of Dermatology, Lund University, Sweden; 4 Department of Dermatology and Venereology, Institute of Clinical Science, Sahlgrenska Academy, University of Gothenburg, Gothenburg, Sweden; 5 Region Västra Götaland, Sahlgrenska University Hospital, Department of Dermatology and Venereology, Gothenburg, Sweden

**Keywords:** skin cancer, melanoma, risk factors, sun habits

## Abstract

**Background:**

Skin cancer incidence is rapidly increasing. The main risk factor, sun exposure, can be modified. Informational campaigns can be effective in raising skin cancer awareness and target the high-risk population. Still, sun exposure habits in people at high risk of skin cancer are not well-known.

**Objective:**

To investigate if and how sun exposure habits differ between low-risk and high-risk individuals.

**Methods:**

During the Swedish Euromelanoma campaign of 2018, questionnaires were collected containing information regarding sun exposure habits and risk factors for skin cancer. Data on 4,141 participants was used to investigate the association between risk factors and sun exposure habits.

**Results:**

A fair skin type and a previous history of skin cancer were significantly associated with enhanced sun protective behavior. Family history of skin cancer, childhood sunburns and the presence of large/atypical nevi had no effect on sun exposure habits. Going on sunny holidays were particularly unaffected by being at high risk of skin cancer.

**Conclusion:**

Individuals at high risk of developing skin cancer showed suboptimal sun exposure habits and harmful traveling behaviors. We suggest that future skin cancer campaigns inform on accurate sun protection behavior during sunny holidays and associated risk factors. Risk factors such as childhood sunburns, numerous common and large/atypical nevi, as well as family history of skin cancer seem to be less recognized by the population.

## Introduction

Skin cancer is the most common cancer worldwide, predominantly affecting countries with mainly fair-skinned individuals such as Australia, New Zealand, North America, and Europe [1]. Compared to overall cancer incidence, which has decreased globally in recent years [2], skin cancer incidence is rapidly increasing [3, 4]. This trend has also been observed in Sweden where the incidence of the 3 main subtypes of skin cancer (malignant melanoma, MM; squamous cell carcinoma, SCC, and basal cell carcinoma, BCC) are increasing at a fast pace, of approximately 5–6% per year [5].

MM is the most aggressive form of skin cancer, accounting for 90% of all skin cancer mortality [1, 6]. The most important risk factor for MM is ultraviolet (UV) radiation which can be attributed to 65–90% of all MM cases [7, 8]. Other important risk factors for MM are fair skin types (I–II according to Fitzpatrick), many common nevi, multiple large/atypical nevi, childhood sunburns, family history of MM, and personal history of skin cancer. SCC and BCC derive from keratinocytes and are often referred to as types of keratinocyte cancer (KC). KCs are very common and may cause significant morbidity but rarely metastasize [9, 10].

Due to the modifiability of the main risk factor for skin cancer, UV radiation, interest to influence people to adopt healthier sun exposure habits has been raised. This can be accomplished through skin cancer awareness campaigns [11–15]. To optimize these campaigns, it is important to have knowledge of sun exposure behavior within the population. Nevertheless, there is scarce evidence on differences in sun exposure habits between individuals with high risk and low risk of skin cancer. 2 studies have shown that fair-skinned individuals have safer sun exposure habits than dark-skinned individuals [16, 17], but one Italian study found the opposite relationship [18]. A Swedish cross-sectional study suggested that a personal history of skin cancer induced sun protective habits [19], but a systematic review from 2015 on individuals with a history of KC found that their sun protective behavior continued to be suboptimal [20]. Using data from the 2018 Swedish Euromelanoma week campaign for skin cancer awareness, we aimed to investigate sun exposure habits in people at different risk of skin cancer.

## Methods

The Regional Ethical Review Board in Gothenburg, Sweden waived the need for ethical approval (registration number 608-08) since the data was anonymised.

### Data collection

Every year since 1999, a European-wide skin cancer campaign, named Euromelanoma, is carried out in over 30 European countries [21]. During the campaign, individuals are screened for skin cancer. The studied Euromelanoma week, was held during May 14–18, 2018. Dermatology clinicians from all over Sweden were invited to participate. Every patient participating in the Euromelanoma week filled in a 2-page questionnaire (Supplementary material 1). The first page of the questionnaire was completed by the patient and contained questions on sun habits, sun sensitivity, severe childhood sunburns, outdoor occupation, sunny holidays, and current use of sunbeds. The second page, completed by the dermatologist, included information regarding family history of skin cancer, patient history of skin cancer, number of common and large/atypical nevi (defined as asymmetric with ill-defined border, irregular pigmentation/color and a diameter >6mm), presence of actinic keratoses, lentigines, and clinically suspected skin cancers.

### Statistical Analysis

Firstly, univariate associations between risk factors for skin cancer and sun exposure behavior were tested. Secondly, we constructed a multinomial regression model to test the odds of choosing one sun exposure behavior over another in the different risk categories. Lastly, to compare people with varied combinations of risk factors for MM, we designed a score for different levels of risk for MM. Risk factors that were used in the score were: skin type, number of common nevi, number of large/atypical nevi, family history of MM, personal history of skin cancer, and severe childhood sunburns. To correctly weigh risk factors, we used risk estimates from published meta-analyses. These were found by a literature search on PubMed using the keywords “malignant melanoma AND risk factor” and “skin cancer AND risk factor”. We included studies in English and published between 1998–2018, which yielded 6 meta-analyses (Supplementary material 2) [22–27]. Since relative risks are multiplicative, the logarithms of the risk estimates from the meta-analyses were summed to create a score on the linear scale. This yielded a score ranging from 0–7. Cut-offs (≤0.5, >0.5–2, >2) were chosen to clearly separate out people with a high-risk score and a low-risk score from the majority in the normal distribution of the risk-score. Randomized non-parametric chi-χ^2^-tests were used to test significance, set to p<0.05 [28, 29].

## Results

**Figure 1:**
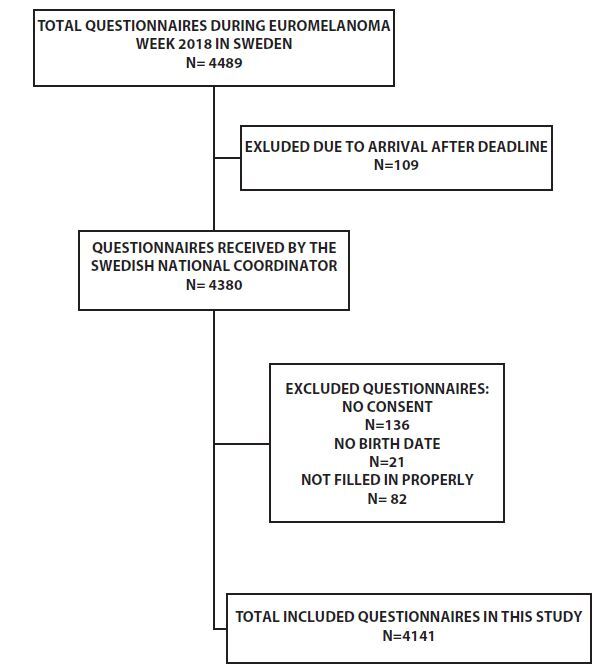
Flow-chart for inclusion of questionnaires from the Swedish Euromelanoma week 2018.

43 dermatology clinics enrolled for participation and offered 5,521 appointments all over Sweden. A total of 4,489 questionnaires were received. After exclusions due to incomplete information on date of birth (n=21), no written consent (n=136), or no information entered on one of the two pages (n=191), 4,141 questionnaires (Figure 1) were left for analyses. Overall, each question had a high answer rate of over 95%.

Most participants were women (66%), and 58% had a university degree. Self-assessment of skin type was as follows: 3% skin type I, 18% skin type II, 57% skin type III and 22% skin type IV. The vast majority (96%) reported not using sunbeds and 12% not to sunbathe. Most participants (59%) stated always using sunscreen when sunbathing and 87% reported going on sunny holidays every year. 9% of patients had a family history of MM. A personal history of skin cancer was found in 11% of patients (8% KC and 3% MM). For detailed descriptive statistics, see (Supplementary material 3).

### Risk Behavior in Relation to Sex and Age

Men were 40% more likely to not sunbathe compared to women and at the same time 50% more likely to never apply sunscreen while sunbathing. There was no difference in travelling habits between men and women. With increasing age, the participants reported less sunbathing, sunscreen use and less sunny holidays (Table 1).

### Risk Behavior in Relation to Skin Type

Skin type was associated with all measured sun behaviors. Multivariate analysis showed that people with skin type II and skin types III–IV, respectively, were 70 and 90% more likely to sunbathe compared to people with skin type I. Likewise, people with skin types II–IV were only half as likely to always, rather than sometimes, use sunscreen when sunbathing compared to people with skin type I. Going on sunny holidays for ≤ 2 weeks/year (compared to no sunny holidays) was 2.3 times more common in people with skin type II and 3.5 times more common in people with skin types III–IV. This effect was even more pronounced when looking at the odds of going on sunny holidays for > 2 weeks/year (Table 2).

### Risk Behavior in Relation to Childhood Sunburn and Family History of Melanoma

A higher proportion of people who remembered having had a childhood sunburn reported not to sunbathe and to always use sunscreen when sunbathing, compared to people who did not remember such an event. However, this effect disappeared in the multivariate analyses. No other sun exposure behaviors were associated with childhood sunburns.

A positive family history of MM was only associated with sunscreen use when sunbathing (p=0.03) in the univariate analyses, but even this association disappeared in the adjusted analyses (Table 3).

### Risk Behavior in Relation to History of Skin Cancer

Having had MM or KC increased the odds of choosing not to sunbathe by 2.5 and 1.5 times, respectively, compared to an individual with no skin cancer history. The odds of always, rather than sometimes, applying sunscreen when sunbathing increased by 2.2 times in people with a history of MM and 1.5 times in people with a history of KC, compared to people with no history of skin cancer. Travelling behavior was almost unaffected by having had a skin cancer (Table 4).

### Risk Behavior in Relation to Nevi

Not sunbathing was only significantly associated with having 25–50 common nevi (but not to >50 nevi) compared to <25 nevi. To always use sunscreen when sunbathing was more clearly associated with the number of common nevi, with 20%, 30% and 40% odds increases in people with 25–50, 50–100 and >100 common nevi, respectively. Going on sunny holidays was not associated with the number of common nevi (Table 5). The presence of large/atypical nevi did not affect sun exposure habits (data not shown).

### Risk Behavior in Relation to Risk Score

The melanoma risk score yielded 416 individuals with a low-risk score, 2886 with a medium-risk score, and 839 with a high-risk score. There was a higher proportion of women (68% vs 61%), a lower mean age (53 vs 63 years), and a higher educational level (61% vs 42% with a university degree) in the high-risk compared to the low-risk score group.

People with a high-risk score were almost 2 times as likely not to sunbathe and more than 2 times as likely to use sunscreen when sunbathing compared to people with a low-risk score. Going on sunny holidays was not affected by risk score group (Table 6).

## Discussion

Based on data from questionnaires obtained during the Swedish Euromelanoma week campaign of 2018, sun exposure habits in individuals with different risk factors for developing skin cancer were compared. Skin type affected sun exposure habits the most. Age, sex, and having had a previous skin cancer was also somewhat associated with sun exposure behavior. A family history of skin cancer, the presence of large/atypical nevi, and childhood sunburns had no effects on sun exposure habits. Frequency of going on sunny holidays was only affected by having a fair skin type and, modestly, by age.

In agreement with earlier studies, women reported to sunbathe and to use sunscreen while sunbathing more frequently, compared to men [30, 31]. This “sunscreen paradox” in which sunscreen use opens up for prolonged sun exposure has been described before [32]. Such an incorrect use of sunscreens, may result in an increased risk of skin cancer [32]. Skin cancer campaigns can help to raise awareness of this potential snare and induce healthier sun exposure habits [11, 12]. As in prior health care interventions and campaigns, most of the participants in our study were women [33, 34]. Considering that men in general have less healthy sun habits [16, 31] and a poorer prognosis of MM [35], it is important to design future campaigns to better capture their attention. High age (>70 years) has previously been associated with lower awareness of MM risk factors [36]. This is supported by our finding that participants reported less use of sunscreen with increasing age. However, this might partly be secondary to the decreased sunbathing and travelling on sunny holidays with older age.

Patient-estimated skin type was a strong predictor of sun protective behavior in our study as well as others [16, 17, 31]. This might be explained by the instant negative feedback, in form of sunburns, that individuals of skin types I and II experience when sunbathing. Nonetheless, 40% of individuals with skin type I and 80% of individuals with skin type II answered that they sunbathe, and the majority stated that they went on sunny holidays every year (70 and 80%, respectively). These results also indicate that information about adjusting sun exposure level to the individual presupposition should be emphasized in future skin cancer campaigns.

Severe childhood sunburns did not significantly affect any sun protective behavior. This may indicate a low awareness in the general population of the heightened skin cancer risk that severe childhood sunburns convey [23]. However, since almost 30 % answered that they “do not remember” if they experienced a severe sunburn before 18 years of age, the results might be afflicted by recall bias, or at least non-differential information bias, making it hard to draw any solid conclusions from them. A low awareness of childhood sunburns in Sweden, Denmark, Norway and Northern England has also been found in a prior study [36].

Having a first-degree relative with a previous MM had no impact on the measured sun exposure behaviors. This study therefore adds weight to the findings of previous studies that sun protective behavior is still suboptimal in family members to persons afflicted by MM [37–40]. A possible explanation is that individuals with a family history of MM might have “inherited” a risk behavior in childhood that is difficult to change later in life [38, 41]. The sun exposure behavior was, however, safer in people who themselves had had skin cancer in this study. Nevertheless, it should be noted that 20% and 27% of people with a history of MM and KC, respectively, still sunbathe and that travelling behavior was nearly unaffected by having had a skin cancer. The suboptimal sun exposure behaviors in people with a history of skin cancer has also been documented previously [19, 20, 42]. A Danish prospective case–control study indicated that people diagnosed with MM did improve their sun protective behavior, but only temporarily [42].

Having many common nevi was associated with safer sun exposure behavior, mainly characterized by sunscreen use while sunbathing. However, the presence of large/atypical nevi had no effect on sun exposure behavior. We know from previous studies that the number of nevi is associated with the degree of sun exposure in childhood [43, 44] and also to risk of MM [22]. Our results indicate that these associations are either not well-known or neglected in the general population. As the presence of many common nevi as well as the presence of large nevi are objective factors, easily observed by patients themselves, these might represent important risk factors that should be highlighted in future skin cancer awareness campaigns.

Our constructed risk score reflects an individual’s combined risk for skin cancer by weighing in all measured risk factors in 1 variable. Indeed, we found lower odds of sunbathing and higher odds of using sunscreen while sunbathing in the group with the highest risk score for skin cancer, but we found no association with going on sunny holidays. However, differences in the risk score groups were not impressive and a high proportion of people with a high-risk score still had suboptimal sun exposure behavior.

The observed unhealthy sun exposure habits in people at risk for skin cancer can have several explanations. A Swedish study examining attitudes and subjective norms predicting sun protective behavior found an association between positive attitudes to getting a tan or being in the sun with UV exposure behaviors such as intentional tanning, sunbed use and the frequency of sunny holidays. The study also showed that group pressure affected sun exposure behavior [45]. A systematic review of 23 studies showed similar results and key motivators for sun exposure behaviors identified were perception of appearance and health and influence of parents, peers and media [41]. The ideal “tanned look” that prevails in modern society and bolstered by media might be another explanation [45]. Furthermore, studies have shown that “UV-seekers” meet diagnostic criteria for substance-related disorders, with regards to UV exposure [46, 47]. Another study performed in rodents, showed that ß-endorphins (endogenous opioid neuropeptides and peptide hormones that block pain signaling and produce a feeling of euphoria/pleasure) are synthesized at the level of the skin and elevated in plasma following low-dose UV exposure [48]. Hence, there might be elements of physiological addiction to sun exposure which can both explain observed habits and obstruct a change of sun exposure behavior. Skin cancer incidence is increasing rapidly but can be prevented by changing sun exposure behavior in the general population. From the results in this study, we suggest that skin cancer awareness campaigns should contain additional information on less known or ignored risk factors, such as having a first-degree relative with skin cancer, numerous nevi, multiple large/atypical nevi, or having suffered childhood sunburns. Although the Euromelanoma campaigns have been ongoing since 2000 [49], information about all risk factors and unhealthy sun exposure behaviors must be repeated and especially approached from a different angle, since our study results shed light on a general suboptimal sun exposure behavior in many participants. Furthermore, the risk behavior least affected by being at risk of skin cancer in this study (going on sunny holidays) must be addressed. This finding is supported by 2 previous studies in which a high frequency of sunburns and inadequate sun protection was found in Danes travelling to sunny destinations [50, 51]. Therefore, efforts should be made to emphasize the importance of a sun protective behavior at a UV index ≥ 3, both at home and when travelling [52]. Smartphone applications (e.g. “Min soltid”/“My sun time” launched by the Swedish Meteorological and Hydrological Institute in cooperation with the Swedish Radiation Safety Authority) that calculate a safe sun exposure time based on UV index and skin type might be an interesting focus of future skin cancer prevention campaigns [53].

The strengths of this study are the large size and the contemporary data from 2018. The information was collected from the entire country on people of both genders, a wide age range, and all levels of education. However, our study has also several weaknesses. Firstly, all information regarding sun exposure was self-reported and might be affected by self-assessment bias (eg skin type). We could, however, not detect a difference in self-assessed skin type with sex or level of education. Secondly, some of the questions from the questionnaire were phrased inadequately, such as question 11.1 “Number of weeks per year at sunny holidays” which did not include a definition of what was meant by a sunny holiday. Consequently, this question could have been interpreted differently by participants, possibly introducing non-differential information bias. Lastly, the distribution of levels of education and gender of the participants is not representative of the entire Swedish population. This might affect the ability to generalize our results but does not affect the internal validity.

In summary, raising awareness of the risk factors that had no impact on sun exposure behavior (family history of MM, presence of large/atypical nevi and childhood sunburn) in this study, could be an important part of future skin cancer awareness campaigns. Also, sun exposure habits during sunny holidays should be specifically addressed. Finally, the underlying psychological reasons for continuing a suboptimal sun exposure behavior should be exposed and, if possible, influenced.

## Supplementary Material

Supplementary material 1 Euromelanoma questionnaire (Figure S1).

Supplementary material 2 Meta-analyses of known risk factors for malignant melanoma and estimated risk score. The sum of the log of the relative risk was used to create the score values for each individual, stating their combined risk increase for malignant melanoma (Table S2).

Supplementary material 3 Association between specific risk factors and sun exposure habits. Statistical significance was tested with chi-square tests (Table S3).

## Figures and Tables

**Table 1:** Multivariate Odds Ratios (OR) of Choosing a Risk Behavior (use of sunscreen when sunbathing, sunbathing and going on sunny holidays) With Regard to Age and Sex, Using Female as Reference Category and for Every 10-Year Increase in Age.

	Female	Male	Age (odds ratio for every 10-year increase)

**I never sunbathe**			
False		*Reference behavior*	*Reference behaviour*
True	*Reference category*	**1.4 (1.2–1.8)**	**1.3 (1.2–1.4)**

**Use of sunscreen when sunbathing**			
Never	*Reference category*	**1.5 (1.0–2.3)**	**1.3 (1.2–1.5)**
Sometimes		*Reference behavior*	*Reference behavior*
Always		**0.5 (0.4–0.5)**	**0.9 (0.8–0.9)**

**Number of weeks at sunny holidays/year**			
0	*Reference category*	*Reference behavior*	*Reference behavior*
≤2 weeks/year		0.9 (0.7–1.1)	**0.8 (0.7–0.8)**
>2 weeks/year		0.9 (0.7–1.1)	**0.8 (0.7–0.9)**

OR= odds of choosing a behavior over the reference behavior, compared to the same choice of behavior in the reference category (female sex). The estimates for age reflect the difference in choice of a behavior for every 10-year increase in age. Estimates adjusted for skin type, childhood sunburn, family history of melanoma, personal history of skin cancer, number of nevi. Significance level set to P < 0.05.

**Table 2:** Multivariate Odds Ratios (OR) of Choosing a Risk Behavior (use of sunscreen when sunbathing, sunbathing, and going on sunny holidays) With Regard to Skin Type, Using Skin Type I as Reference Category

	Skin type I	Skin type II	Skin type III	Skin type IV

**I never sunbathe**				
False	*Reference category*	*Reference behavior*	*Reference behavior*	*Reference behavior*
True		**0.3 (0.2–0.5)**	**0.1 (0.1–0.2)**	**0.1 (0.1–0.2)**

**Use of sunscreen when sunbathing**				
Never		**0.5 (0.3–0.9)**	0.9 (0.5–1.5)	1.1 (0.6–1.9)
Sometimes		*Reference behavior*	*Reference behavior*	*Reference behavior*
Always	*Reference category*	**0.5 (0.3–0.8)**	**0.4 (0.2–0.6)**	**0.4 (0.2–0.6)**

**Number of weeks at sunny holidays/year**				
0	*Reference category*	*Reference behavior*	*Reference behavior*	*Reference behavior*
≤2 weeks/year		**2.3 (1.4–3.9)**	**3.5 (2.2–5.8)**	**3.4 (2.0–5.8)**
>2 weeks/year		**2.4 (1.4–4.3)**	**5.0 (2.9–8.6)**	**6.0 (3.4–10.8)**

OR= odds of choosing a behavior over the reference behavior, compared to the same choice of behavior in the reference category (skin type I). Estimates adjusted for age, sex, childhood sunburn, family history of melanoma, personal history of skin cancer, number of nevi. Significance level set to P < 0.05.

**Table 3:** Multivariate Odds Ratios (OR) of Choosing a Risk Behavior (use of sunscreen when sunbathing, sunbathing and going on sunny holidays) With Regard to Family History of Melanoma, Using no Family History of Melanoma as Reference Category.

	No family history of melanoma	One first-degree relative with history of melanoma	Two or more first-degree relatives with history of melanoma

**I never sunbathe**			
False	*Reference category*	*Reference behavior*	*Reference behavior*
True		1.2 (0.9–1.8)	0.9 (0.4–2.0)

**Use of sunscreen when sunbathing**			
Never		0.9 (0.4–2.0)	1.0 (0.1–7.3)
Sometimes	*Reference category*	*Reference behavior*	*Reference behavior*
Always		1.0 (0.7–1.3)	1.3 (0.7–2.3)

**Number of weeks at sunny holidays/year**			
0	*Reference category*	*Reference behavior*	*Reference behavior*
≤2 weeks/year		1.0 (0.7–1.5)	1.1 (0.4–2.7)
>2 weeks/year		1.3 (0.9–2.0)	1.8 (0.7–4.4)

OR= odds of choosing a behavior over the reference behavior, compared to the same choice of behavior in the reference category (no family history of melanoma). Estimates adjusted for age, sex, skin type, childhood sunburn, personal history of skin cancer, number of nevi. Significance level set to P < 0.05.

**Table 4:** Multivariate Odds Ratios (OR) of Choosing a Risk Behavior (use of sunscreen when sunbathing, sunbathing and going on sunny holidays) With Regard to Personal History of Skin Cancer (malignant melanoma (MM) or keratinocyte cancer (KC)), Using no History of Skin Cancer as Reference Category.

	No history of skin cancer	History of MM	History of KC

**I never sunbathe**			
False	*Reference category*	*Reference behavior*	*Reference behavior*
True		**2.5 (1.5–4.1)**	**1.47 (1.1–2.1)**

**Use of sunscreen when sunbathing**			
Never		1.9 (0.6–5.8)	1.0 (0.5–2.0)
Sometimes	*Reference category*	*Reference behavior*	*Reference behavior*
Always		**2.2 (1.2–3.8)**	**1.6 (1.2–2.1)**

**Number of weeks at sunny holidays/year**			
0	*Reference category*	*Reference behavior*	*Reference behavior*
≤2 weeks/year		0.7 (0.4–1.3)	0.8 (0.6–1.2)
>2 weeks/year		**0.4 (0.2–0.7)**	0.9 (0.6–1.3)

OR= odds of choosing a behavior over the reference behavior, compared to the same choice of behavior in the reference category (no history of skin cancer). Estimates adjusted for age, sex, skin type, childhood sunburn, family history of melanoma, number of nevi. Significance level set to P < 0.05.

**Table 5:** Multivariate Odds Ratios (OR) of Choosing a Risk Behavior (use of sunscreen when sunbathing, sunbathing, and going on sunny holidays) With Regard to Number of Common Nevi, Using <25 Common Nevi as Reference Category.

	<25 common nevi	25–50 common nevi	50–100 common nevi	>100 common nevi

**I never sunbathe**				
False	*Reference category*	*Reference behavior*	*Reference behavior*	*Reference behavior*
True		**0.7 (0.6–0.9)**	0.8 (0.5–1.1)	0.6 (0.3–1.2)

**Use of sunscreen when sunbathing**				
Never	*Reference category*	0.8 (0.5–1.3)	0.9 (0.4–1.8)	1.0 (0.3–3.3)
Sometimes		*Reference behavior*	*Reference behavior*	*Reference behavior*
Always		**1.2 (1.0–1.5)**	**1.3 (1.0–1.7)**	**1.4 (1.0–2.2)**

**Number of weeks at sunny holidays/year**				
0	*Reference category*	*Reference behavior*	*Reference behavior*	*Reference behavior*
≤2 weeks/year		1.2 (1.0–1.6)	1.1 (0.8–1.5)	1.2 (0.7–2.2)
>2 weeks/year		1.0 (0.8–1.3)	1.1 (0.8–1.54)	0.8 (0.4–1.4)

OR= odds of choosing a behavior over the reference behavior, compared to the same choice of behavior in the reference category (<25 common nevi). Estimates adjusted for age, sex, skin type, childhood sunburn, family history of melanoma, personal history of skin cancer. Significance level set to P < 0.05.

**Table 6:** Multivariate Odds Ratios (OR) of Choosing a Risk Behavior (use of sunscreen when sunbathing, sunbathing, and going on sunny holidays) With Regard to Risk Score, Using Low-Risk Score as Reference Category.

	Low-risk score	Medium-risk score	High-risk score

**I never sunbathe**			
False	*Reference category*	*Reference behavior*	*Reference behavior*
True		1.1 (0.8–1.5)	**1.8 (1.2–2.5)**

**Use of sunscreen when sunbathing**			
Never	*Reference category*	0.7 (0.5–1.1)	0.56 (0.3–1.1)
Sometimes		*Reference behavior*	*Reference behavior*
Always		**1.6 (1.2–2.0)**	**2.3 (1.8–3.0)**

**Number of weeks at sunny holidays/year**			
0	*Reference category*	*Reference behavior*	*Reference behavior*
≤2 weeks/year		1.2 (0.9–1.7)	1.0 (0.7–1.5)
>2 weeks/year		0.9 (0.7–1.3)	0.7 (0.5–1.0)

OR= odds of choosing a behavior over the reference behavior, compared to the same choice of behavior in the reference category (low-risk score). Estimates adjusted for age and sex. Significance level set to P < 0.05.
