# Supplementary material for: Difference in Sun Exposure Habits Between Individuals with High and Low Risk of Skin Cancer
Source: Dermatol Pract Concept. 2021 Oct 1;11(4):e2021090. doi: 10.5826/dpc.1104a90 (PMC8480439; doi:10.5826/dpc.1104a90)
Supplement: Supplementary material 1 — Euromelanoma questionnaire (Figure S1). [file dp1104a90s1.pdf]

Dermatology Practical & Conceptual

Difference in Sun Exposure Habits Between Individuals with High and Low Risk of Skin Cancer

Oskar Karlsson, Oskar Hagberg, Kari Nielsen, John Paoli, Åsa Ingvar

Supplementary Material

Figure S1. Euromelanoma Questionnaire

EUROMELANOMA QUESTIONNAIRE

To be completed by person screened:

0. I acknowledge that the information on this sheet and the results of any tissue sample are used for compiling statistics for follow-up of the campaign and for research (the information is de-identified before compiling data). The de-identified data will be compiled into a European database including data from several countries: No ☐ Yes ☐

1. Gender: Male ☐ Female ☐

2. Date of Birth: (day/month/year) _ _ / _ _ / _ _ _ _

3. What is Your highest degree of education?

☐ Primary school ☐ High school ☐ Vocational education ☐ University degree

4. Why did you participate in Euromelanoma? (Tick all that apply)

☐ I have many moles
☐ Recently changed or suspicious lesion
☐ I was previously diagnosed with a skin cancer
☐ I have a family member or friend with skin cancer
☐ Because I want to have my skin checked

5. Have you previously received a full skin examination? (including Euromelanoma)

No ☐ Yes ☐ __ Number of times

6. Did or do you have an outdoor occupation? If yes, for how many years?

☐ No
☐ Yes, for:
☐ 1 year or less
☐ more than 1, until 5 years
☐ more than 5, until 10 years
☐ more than 10 years

7. How does your skin react to the summer sun?

☐ My skin always burns, never tans
☐ My skin always burns, tans minimally or with difficulty
☐ My skin initially burns and then tans
☐ My skin burns minimally, tans readily

8. Did you suffer from severe sunburn (a painful sunburn, with intense redness or blistering, lasting for 2 days or more) before the age of 18?

☐ No
☐ Yes
☐ I don’t remember

9. How often do you use sunscreens when you are exposed to the sun?

9.1. When you are outdoor for > 1 hour (other than sunbathing):
☐ Never
☐ Sometimes
☐ Always

9.2. Do you apply sunscreen when you are sunbathing:
☐ Never
☐ Sometimes
☐ Always

9.3. I never take a sunbath: ☐

10. Did you spend in total one year or more in a country with much higher sun exposure than the country where you currently live?
☐ No
☐ Yes, before the age of 18: ☐☐ years
☐ Yes, after the age of 18: ☐☐ years

11. Sun exposure during adulthood?

11.1. Number of weeks per year at sunny holidays:
☐ 0
☐ 2 weeks or less
☐ more than 2 weeks

11.2. Do you use solarium?
☐ No
☐ Yes 20 sessions or less/year
☐ Yes, 21 or more sessions/ year

11.3. Number of years using solarium (including in the past only): ☐☐ yrs

To be completed by physician:
12. Family history of melanoma (melanoma in first degree relatives: father, mother, brother and sister):
☐ No
☐ Yes:1 relative
☐ Yes: >2 first degree relatives
☐ Patient doesn’t know

13. Personal history of skin cancer:
☐ No
☐ Yes, melanoma
☐ Yes, non-melanoma skin cancer
☐ Patient doesn’t know if he/she has had skin cancer

14. Skin examination performed today:
☐ full
☐ partial

15. I used dermoscopy to examine this patient:
☐ Yes
☐ No

16. Clinical examination:
☐ Yes
☐ No

16.1. Number of moles:
☐ <25
☐ 25-50
☐ 50-100
☐ >100

16.2 Presence of lentigines on the back / chest:

16.3 Presence of atypical moles (according to definition*):
* (asymmetry, ill-defined border, irregular pigmentation/color, diameter >6mm)

16.4 Presence of actinic keratoses:

17. Clinically suspicious lesions #:
☐No ☐Yes
☐No ☐Yes Number: ___
☐No ☐Yes

1. Melanoma: ☐ No * ☐ Yes __ Number

2. 2. BCC: ☐ No + ☐ Yes __ Number
   3. SCC: ☐ No # ☐ Yes __ Number

4. Other or clinically undefined: ☐ No ☐ Yes __ Number

18. The lesion was first detected by (please fill only when there is a clinically suspicious lesion observed by the dermatologist, if there are several lesions, the clinically most important one):
☐ patient
☐ dermatologist
☐ another health professional
☐ spouse/partner
☐ other person
