# Supplementary material for: Difference in Sun Exposure Habits Between Individuals with High and Low Risk of Skin Cancer
Source: Dermatol Pract Concept. 2021 Oct 1;11(4):e2021090. doi: 10.5826/dpc.1104a90 (PMC8480439; doi:10.5826/dpc.1104a90)
Supplement: Supplementary material 2 — Meta-analyses of known risk factors for malignant melanoma and estimated risk score. The sum of the log of the relative risk was used to create the score values for each individual, stating their combined risk increase for malignant melanoma (Table S2). [file dp1104a90s2.pdf]

Dermatology Practical & Conceptual

Difference in Sun Exposure Habits Between Individuals with High and Low Risk of Skin Cancer

Oskar Karlsson, Oskar Hagberg, Kari Nielsen, John Paoli, Åsa Ingvar

Supplementary Material

Table S2. Meta-analyses of known risk factors for malignant melanoma and estimated risk score. The sum of the log of the relative risk was used to create the score values for each individual, stating their combined risk increase for malignant melanoma.

Study	Risk factor categorization in meta-analyses	Relative risk for melanoma	Risk factor categorization in the analyses of this study	Converted risk score
Gandini et al 2005	Skin type		Skin type	
	I	2.09 RR	I	2.09
	II	1.84 RR	II	1.84
	III	1.77 RR	III	1.77
	IV	1.00 RR	IV	1.00
Gandini et al 2005	Number of common nevi		Number of common nevi	
	0–15	1.00 RR	<25<br>25-50<br><br>50-100	1.00<br>1.86<br><br>3.41
	16–40	1.47 RR		
	41–60	2.24 RR		
	61–80	3.26 RR		
	81–100	4.74 RR		
	101–120	6.89 RR	>100	6.89
Gandini et al 2005	Number of large/ atypical nevi		Number of large/ atypical nevi	
		1.00 RR	0	1.00
	1	1.45 RR	1	1.45
	2	2.10 RR	2	2.10
	3	3.03 RR	3	3.03
	4	4.39 RR	4	4.39
	5	6.36 RR	5	6.36
Gandini et al 2005	First-degree-relative with melanoma	1.74 RR	First-degree-relative with melanoma	1.74
Van der Leest et al 2015	Personal history of melanoma	10.4 SIR	Personal history of melanoma	10.4
Flohil et al 2013	Personal history of BCC	2.4 SIR	Personal history of KC	2.6
Flohil et al 2013	Personal history of SCC	2.8 SIR		
Gandini et al 2005	Childhood sunburn (before age 15)	2.24 RR	Childhood sunburn	2.1
Dennis et al 2008	Childhood sunburn (before age 20)	1.91 OR		

RR: Relative risk, OR: Odds ratio, SIR: Standardised incidence ratio, BCC: Basal cell carcinoma, SCC: Squamous cell carcinoma, KC: Keratinocyte cancer
