# Supplementary material for: Difference in Sun Exposure Habits Between Individuals with High and Low Risk of Skin Cancer
Source: Dermatol Pract Concept. 2021 Oct 1;11(4):e2021090. doi: 10.5826/dpc.1104a90 (PMC8480439; doi:10.5826/dpc.1104a90)
Supplement: Supplementary material 3 — Association between specific risk factors and sun exposure habits. Statistical significance was tested with chi-square tests (Table S3). [file dp1104a90s3.pdf]

Dermatology Practical & Conceptual

Difference in Sun Exposure Habits Between Individuals with High and Low Risk of Skin Cancer

Oskar Karlsson, Oskar Hagberg, Kari Nielsen, John Paoli, Åsa Ingvar

Supplementary Material

Table S3. Association between specific risk factors and sun exposure habits. Statistical significance was tested with chi-square tests.

		Use of sunscreen when sunbathing N (%)			Never sunbathing N (%)		Sunny holidays per year N (%)		
	Total	Never	Sometimes	Always	Yes	No	0 weeks	≤2 weeks	>2 weeks
**Total**	4141	143 (3,4)	1386 (33.5)	2173 (52.5)	507 (12.2)	3629 (87.7)	529 (12.8)	1930 (46.6)	1511 (36.5)
Missing:		444			5		176		
Sex		***p<0.001***			***p=0.02***		***p=0.34***		
Men	1390 (33.9)	76 (6.2)	604 (49.2)	547 (44.6)	200 (14.4)	1186 (85.6)	190 (14.2)	630 (47.2)	514 (38.5)
Women	2716 (66.1)	67 (2.7)	782 (31.6)	1626 (65.7)	307 (11.2)	2443 (88.8)	339 (12.9)	1300 (49.3)	997 (37.8)
Missing:	35	439			5		171		
Skin type		***p<0.001***			***p<0.001***		***p<0.001***		
I	121 (3.0)	5 (6.8)	15 (20.3)	54 (73.0)	51 (42.1)	70 (57.9)	35 (30.4)	52 (45.2)	28 (24.3)
II	739 (18.2)	9 (1.5)	139 (22.6)	468 (76.0)	150 (20.3)	589 (79.7)	119 (16.8)	378 (53.3)	212 (29.9)
III	2308 (56.9)	69 (3.3)	846 (39.9)	1207 (56.9)	207 (9.0)	2098 (91.0)	264 (11.8)	1099 (49.0)	878 (39.2)
IV	891 (22.0)	55 (6.6)	365 (44.0)	409 (49.3)	99 (10.2)	872 (89.8)	94 (11.2)	368 (43.8)	379 (45.1)
Missing:	82	418			5		153		
Family history of melanoma		p=0.03			***p=0.32***		***p=0.16***		
No	3531 (86.7)	128 (4.0)	1198 (37.9)	1835 (58.1)	455 (12.1)	3309 (87.9)	453 (13.4)	1668 (49.2)	1267 (37.4)
Yes	294 (7.2)	7 (2.2)	106 (32.8)	210 (65.0)	52 (14.0)	320 (86.0)	41 (11.5)	164 (46.1)	151 (42.4)
Missing:	66	419			5		159		
Personal history of skin cancer		***p<0.001***			***p<0.001***		***p<0.001***		
No history of skin cancer	3623 (87.5)	120 (3.7)	1240 (38.2)	1886 (58.1)	414 (11.2)	3288 (88.8)	431 (12.5)	1693 (49.0)	1330 (38.5)
History of KC	321 (77.5)	4 (4.7)	21 (24.7)	60 (70.6)	30 (26.5)	83 (73.5)	25 (23.4)	56 (52.3)	26 (24.3)
History of MM	130 (31.4)	13 (4.7)	93 (33.9)	168 (61.3)	63 (19.6)	258 (80.4)	55 (18.0)	134 (43.8)	117 (38.2)
Missing:	*67*	*486*			*5*		*173*		
**Number of nevi**		p<0.001			p<0.001		p<0.001		
<25	2250 (57.0)	93 (4.7)	800 (40.4)	1089 (54.9)	336 (13.8)	2102 (86.2)	314 (14.8)	979 (46.1)	830 (39.1)
25-50	1084 (27.4)	25 (2.5)	338 (34.3)	623 (63.2)	109 (10.1)	974 (89.9)	121 (11.4)	556 (52.4)	385 (36.3)
50-100	465 (11.8)	14 (3.2)	140 (32.5)	277 (64.3)	49 (10.5)	416 (89.5)	53 (11.8)	221 (49.1)	176 (39.1)

Table S3 continues

Table S3. Association between specific risk factors and sun exposure habits. Statistical significance was tested with chi-square tests *(continued)*.

		Use of sunscreen when sunbathing N (%)			Never sunbathing N (%)		Sunny holidays per year N (%)		
>100	151 (3.6)	4 (2.9)	41 (29.9)	92 (67.2)	13 (8.7)	137 (91.3)	15 (10.3)	88 (60.3)	43 (29.5)
Missing:	*191*	*414*			*5*		*169*		
Large/atypical nevi		***p=0.13***			***p=0.12***		***p=0.61***		
0	3267 (88.8)	111 (3.8)	1105 (37.9)	1698 (58.3)	475 (12.6)	3299 (87.4)	425 (13.6)	1504 (48.2)	1191 (38.2)
1	235 (5.7)	6 (2.8)	79 (37.1)	128 (60.1)	19 (8.1)	216 (91.9)	25 (11.0)	106 (46.5)	97 (42.5)
2	43 (1.0)	3 (7.3)	12 (29.3)	26 (63.4)	4 (9.3)	39 (90.7)	3 (7.1)	25 (59.5)	14 (33.3)
3	21 (0.5)	0 (0.0)	4 (19.0)	17 (81.0)	0 (0.0)	21 (100.0)	2 (10.0)	12 (60.0)	6 (30.0)
4	8 (0.2)	0 (0.0)	0 (0.0)	6 (100.0)	1 (12.5)	7 (87.5)	1 (12.5)	4 (50.0)	3 (37.5)
≥5	59 (1.4)	0 (0.0)	17 (31.5)	37 (68.5)	8 (14.5)	47 (85.5)	6 (11.3)	31 (58.5)	16 (30.2)
missing:	*513*	*380*			*5*		*158*		
